# Interactions between platelet activating factor and eicosanoids during endotoxic shock in anaesthetized pigs

**DOI:** 10.1155/S0962935192000280

**Published:** 1992

**Authors:** T. Mózes, F. J. Zijlstra, J. P. C. Heiligers

**Affiliations:** 1 Department of Traumatology Semmelweis Medical University Peterfy Hospital P.O. Box 76 Budapest 1441 Hungary; 2 Department of Pharmacology Faculty of Medicine and Health Sciences Erasmus University Rotterdam Post Box 1738 Rotterdam 3000 DR The Netherlands

## Abstract

The effects of platelet activating factor (PAF) on eicosanoid release during endotoxic shock was investigated in anaesthetized pigs receiving 5 μg kg^−1^ Escherichia coli endotoxin (LPS) into the superior mesenteric artery over a 60 min period, by measuring plasma levels of a variety of mediators. Fifteen of the 31 animals infused with LPS and not treated with BN 52021, a PAF receptor antagonist, died within 30 min after the commencement of LPS infusion (non-survivors), while the other 16 survived the experimental period of 3 h, though in a state of shock (survivors). No alterations were observed in plasma concentrations of eicosanoids in the non-survivors. A significant, though transient, increase in eicosanoid concentrations occurred only in the survivors. Treatment with BN 52021 (4 mg kg-1, i.v.) injected 5 min prior to LPS infusion, failed to exert any effect on the survival rate. However, pretreatment with BN 52021 prevented circulatory collapse in the survivors and reduced the concentration of cyclooxygenase enzyme products, without affecting LTB_4_ release. Exogenous administration of PAF (0.01 μg kg^−1^) caused hypotension and increased TXB_2_ levels although 6-keto PGF_1α_ and LTB_4_ concentrations were unchanged. The data suggest that prostanoid formation may be secondary to PAF release in circulatory collapse evoked by LPS infusion in survivors, and give further support to the suggestion that PAF prostanoid interaction is important during endotoxic shock. However, their role in early death seems to be negligible, indicating the importance of other mediators.


Mediators of Inflammation, 1, 183–190 (1992)

### Interactions between platelet activating factor and eicosanoids during endotoxic shock in anaesthetized pigs

T. Mózes,[1,CA] F. J. Zijlstra[2] and J. P. C. Heiligers[2]

[1]Department of Traumatology, Semmelweis Medical University, Peterfy Hospital, P.O. Box 76, 1441 Budapest, Hungary;
[2]Department of Pharmacology, Faculty of Medicine and Health Sciences, Erasmus University Rotterdam, Post Box 1738, 3000 DR, Rotterdam, The Netherlands

[CA] Corresponding Author

**THE effects of platelet activating factor (PAF) on eicosanoid release during endotoxic shock was investigated in anaesthetized pigs receiving 5 $\mu g\ kg^{-1}$ *Escherichia coli* endotoxin (LPS) into the superior mesenteric artery over a 60 min period, by measuring plasma levels of a variety of mediators. Fifteen of the 31 animals infused with LPS and not treated with BN 52021, a PAF receptor antagonist, died within 30 min after the commencement of LPS infusion (non-survivors), while the other 16 survived the experimental period of 3 h, though in a state of shock (survivors). No alterations were observed in plasma concentrations of eicosanoids in the non-survivors. A significant, though transient, increase in eicosanoid concentrations occurred only in the survivors. Treatment with BN 52021 (4 mg $kg^{-1}$, i.v.) injected 5 min prior to LPS infusion, failed to exert any effect on the survival rate. However, pretreatment with BN 52021 prevented circulatory collapse in the survivors and reduced the concentration of cyclooxygenase enzyme products, without affecting $LTB_4$ release. Exogenous administration of PAF (0.01 $\mu g\ kg^{-1}$) caused hypotension and increased $TXB_2$ levels although 6-keto $PGF_{1\alpha}$ and $LTB_4$ concentrations were unchanged. The data suggest that prostanoid formation may be secondary to PAF release in circulatory collapse evoked by LPS infusion in survivors, and give further support to the suggestion that PAF prostanoid interaction is important during endotoxic shock. However, their role in early death seems to be negligible, indicating the importance of other mediators.**

**Key words:** BN 52021, eicosanoids, endotoxin, PAF, pigs, shock

### Introduction

Effective treatment for the cardiopulmonary derangements characteristic of septic shock is lacking, although sepsis is of major clinical importance and associated with a high morbidity and mortality.[1,2] Sufficient evidence has accumulated which suggests that lipid mediators (platelet activating factor and eicosanoids) are important mediators of endotoxic or septic shock.[3] There are at least three major lines of evidence supporting a role for lipid mediators in endotoxic/septic shock. Products of the arachidonic acid cascade and PAF can be detected in the circulation at concentrations sufficiently high to be responsible for many of the events observed during endotoxic/septic shock.[3–6] Administration of either eicosanoids or PAF into experimental animals produces pathophysiological events characteristic of the state of shock.[3,5,7,8] Pharmacological studies have shown that appropriate synthesis inhibitors or receptor antagonists of lipid mediators are capable of modifying the course of endotoxic/septic shock.[3,5,6,8,9] Although any of these respective observations does not by itself provide proof for a cause–effect relationship, the data taken together suggest that these lipid mediators may play an important role in the development and maintenance of endotoxic/septic shock. Furthermore, evidence is rapidly accumulating that there may be an intimate relationship between PAF and eicosanoids, and some of the effects of PAF may be mediated via the eicosanoids.[10–12] For example, a mediator role for thromboxane has been proposed in PAF-induced cardiac failure[13] and airway hyperresponsiveness.[14] Leukotrienes have been proposed to mediate, in part, pulmonary vasoconstriction and oedema formation evoked by PAF.[15] Both prostanoids and leukotrienes have been demonstrated to mediate PAF-induced bowel necrosis.[16]

Based on these and our previous findings,[8] we hypothesized that an intimate relationship between PAF and eicosanoids may exist in endotoxic shock, too. The purpose of the present work was to

© 1992 Rapid Communications of Oxford Ltd investigate whether eicosanoid formation is secondary to PAF release and to study the role of eicosanoids in mediating PAF induced haemodynamic events during endotoxic shock in anaesthetized pigs.

### Materials and Methods

*Animals:* After an overnight fast, young female Yorkshire pigs (body weight: 20–24 kg, age: 12–16 weeks) were initially anaesthetized with i.m. injections of ketamine (20 mg $kg^{-1}$) and midazolam (0.25 mg $kg^{-1}$) as well as atropine (0.05 mg $kg^{-1}$). The anaesthesia was maintained throughout the experiment with i.v. sodium pentobarbitone (20 mg $kg^{-1}$ bolus followed by a 20 mg $kg^{-1}$ $h^{-1}$ infusion). A tracheostomy was performed and the animals were ventilated with intermittent positive pressure (Bear 2E adult volume ventilator, Lameris, The Netherlands). Respiratory rate, tidal volume and oxygen to air ratio were adjusted to keep arterial blood gases within normal ranges: pH 7.35–7.45, $90 < pO_2$ mmHg $< 150$, $35 < pCO_2$ mmHg $< 45$. The temperature of the animals was maintained at around 38°C by use of an electric blanket. Catheters were placed via femoral vessels into the aorta and pulmonary artery (Swan-Ganz 7F catheter). A midline laparotomy was performed and the superior mesenteric artery was exposed. A needle (0.5 mm external diameter) connected to a suitable polyethylene tube was inserted directly into the superior mesenteric artery for infusion of LPS or normal saline. Another catheter was inserted into the superior mesenteric vein through a branch vein for collecting blood samples and measurement of mean portal venous pressure. The abdomen was then closed and a Ringer-lactate infusion at a rate of 4 ml $kg^{-1}$ $h^{-1}$ was started. The animals were allowed to remain undisturbed for 30 min to ensure haemodynamic stability. Mean arterial blood pressure, mean pulmonary arterial pressure and mean portal venous pressure were continuously monitored by electromanometers with Statham P23db strai gauges (Hato Rey, PR). Cardiac output was determined intermittently by thermodilution (WTI Computer, The Netherlands). The determinations were performed in triplicate and the results averaged.

*Assay for plasma levels of $TXB_2$, 6-keto $PGF_{1\alpha}$ and $LTB_4$:* Blood samples for eicosanoid determinations were obtained from the superior mesenteric vein. The samples were collected into iced polypropylene tubes containing 20 μl of heparin (5000 U $ml^{-1}$, Thromboquline®, Organon, Oss, The Netherlands) and 50 μl of indomethacin (0.1 mg $ml^{-1}$ in 0.1 M phosphate buffer pH 8). After centrifugation plasma was decanted and stored at −70°C until extraction of eicosanoids, while the red blood cells were suspended in 0.9% saline and were injected back after each blood collection. Details of the radioimmunoassay (RIA) for eicosanoid determination have been reported previously.[17]

*Experimental protocols:*

Group I: Sham operated. Five animals were prepared as described above, except that instead of LPS, normal saline was infused into the superior mesenteric artery. Ten ml of blood was collected from the superior mesenteric vein for eicosanoid determination.

Group II: Sham operated, BN 52021 treated. Four animals were prepared as for Group I. Following control measurements and blood collections BN 52021 (4 mg $kg^{-1}$) was injected intravenously 10 min before the second blood collection. Blood was collected as in Group III.

Group III: LPS-induced shock. Thirty-one pigs were infused with 5 μg $kg^{-1}$ *Escherichia coli* LPS (O111 B4, Serva) into the superior mesenteric artery over a 60 min period and the animals were observed for an additional 120 min period. Blood was collected from the superior mesenteric vein for eicosanoid measurements 10 min before, and 15, 30 and 60 min after starting LPS infusion, and 60 and 120 min after LPS infusion.

Group IV: BN 52021 pretreated, LPS infused. A protocol similar to that just described was followed in 13 animals, except that BN 52021 (4 mg $kg^{-1}$ i.v.) was administered 5 min before the LPS infusion was started.

Group V: Injection of PAF into the superior mesenteric vein. In four pigs PAF (1-O-hexadecyl-2-acetyl-*sn*-glycero-3-phosphocholine, Sigma Chemicals, Deisenhofen, Germany) (0.01 μg $kg^{-1}$) was injected into the superior mesenteric vein over 1 min in the absence and presence of BN 52021 (4 mg $kg^{-1}$) administered 5 min prior to PAF injection. Hypotensive response to exogenous PAF and eicosanoid release were tested 10 min before, and 5 min and 60 min after PAF injection.

*Statistical analysis:* All values are expressed as means ± SEM. The data were evaluated by a non-parametric two-way analysis of variance (Friedman test) followed by the Wilcoxon—Wilcox test to identify differences between measurements performed at the control period and at different times during and after LPS infusion. The Mann—Whitney U test was performed to evaluate the differences between the groups. A $p$ value of 0.05 or less was considered statistically significant for all tests.

### Results

*Sham operated animals:* In sham operated animals systemic haemodynamic variables (Figure 1) and eicosanoid levels (Figure 2) were stable during the experiment. Similarly, haemodynamic variables and eicosanoid levels remained stable during the experiments in sham operated animals treated with BN 52021 (Figures 1 and 2).

*Animal survival:* A total of 44 pigs received LPS infusion for 60 min (Groups III and IV). Group III consisted of 31 animals which received LPS only. Fifteen of these 31 animals died at, or close to, 30 min after the start of LPS infusion (non-survivors). The remaining 16 animals survived the experimental period of 3 h (i.e. 2 h after the termination of LPS infusion) though in a state of profound shock (survivors). Treatment with 4 mg $kg^{-1}$ of BN 52021, injected 5 min prior to LPS infusion, failed to exert any effect on the survival rate. Seven of the 13 pigs treated with PAF receptor antagonist died at about 30 min, similar to animals in the LPS treated group. However, the group of survivors treated with BN 52021 resulted in maintenance of most of the parameters at a level comparable to that before LPS infusion.

*Systemic haemodynamics (Figure 1):* In Group III pigs not surviving the LPS infusion, mean arterial blood pressure dropped to 29 ± 2 mmHg, mean portal venous pressure increased to 13.5 ± 1.5 mmHg. Mean pulmonary arterial pressure increased to 44.5 ± 4 mmHg and cardiac output decreased to 0.93 ± 0.11 l/min, shortly before death (30 min after the start of LPS infusion). In BN 52021 pretreated non-survivors similar effects were observed.

In pigs surviving the LPS infusion, mean arterial blood pressure gradually decreased to 59 ± 5.5 mmHg at the end of the 3 h observation period (2 h after the termination of LPS infusion); mean portal venous pressure first increased to peak values at 30 min before decreasing slightly but remaining higher than the baseline values; mean pulmonary arterial pressure increased to a peak value at 30 min before

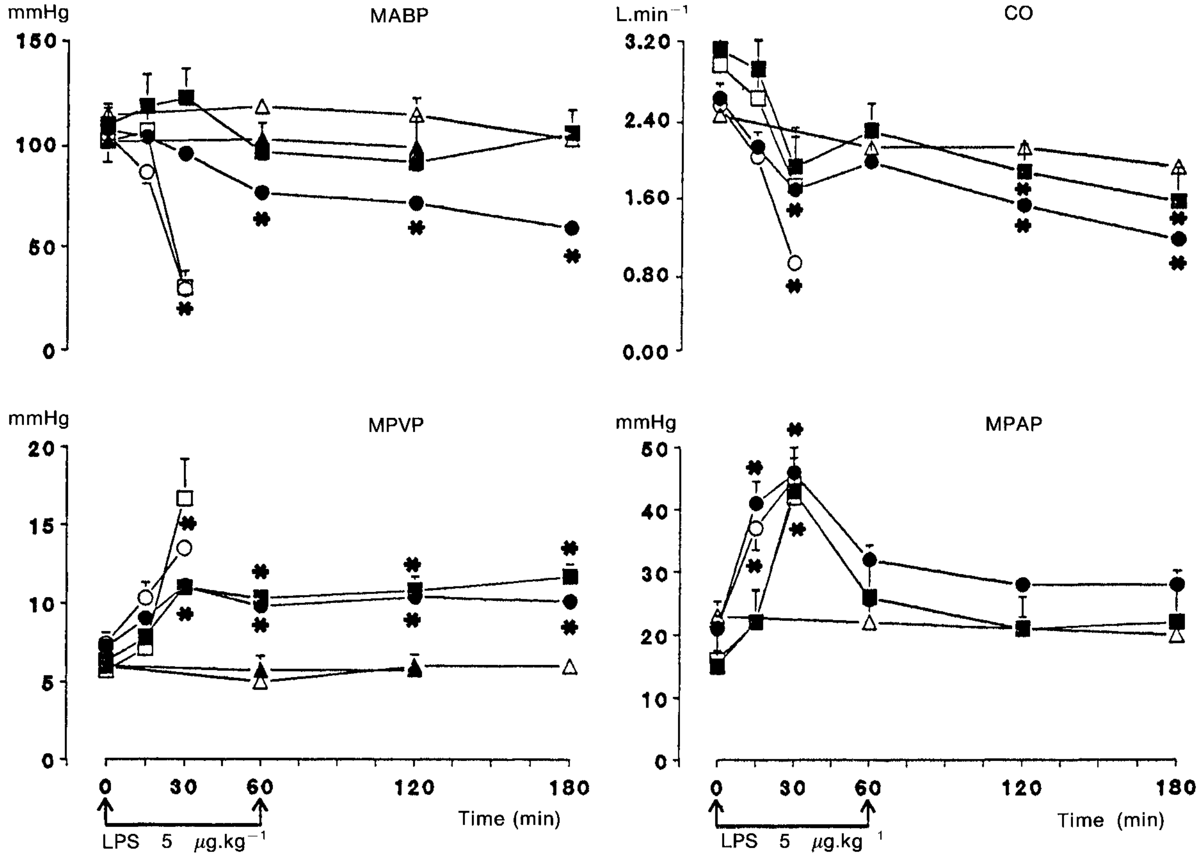

FIG. 1. Effect of BN 52021 (4 mg $kg^{-1}$) pretreatment on systemic haemodynamics in pigs receiving endotoxic (LPS) infusion into the superior mesenteric artery. Arrows indicate the time for administration of LPS. Open triangle indicates sham operated animals ($n = 5$). Closed triangle indicates sham operated, BN 52021 pretreated animals ($n = 4$). Open circle indicates those animals that died within 30 min after starting LPS infusion (non-survivors, $n = 15$). Closed circle indicates those animals that survived the observation period of 3 h after starting LPS infusion (survivors, $n = 16$). Open square indicates BN 52021 pretreated (5 min prior to LPS infusion) non-survivors ($n = 7$). Closed square indicates BN 52021 pretreated survivors ($n = 6$). Values are means with SEM shown by vertical bars. Abbreviations: MABP, mean arterial blood pressure; MPVP, mean portal venous pressure; MPAP, mean pulmonary arterial pressure; CO, cardiac output. *$p < 0.05$ value was obtained by two-way analysis of variance (Friedman test) followed by Wilcoxon—Wilcox test for multiple comparisons, control ($t = 0$ min), control *vs.* 30 min etc.

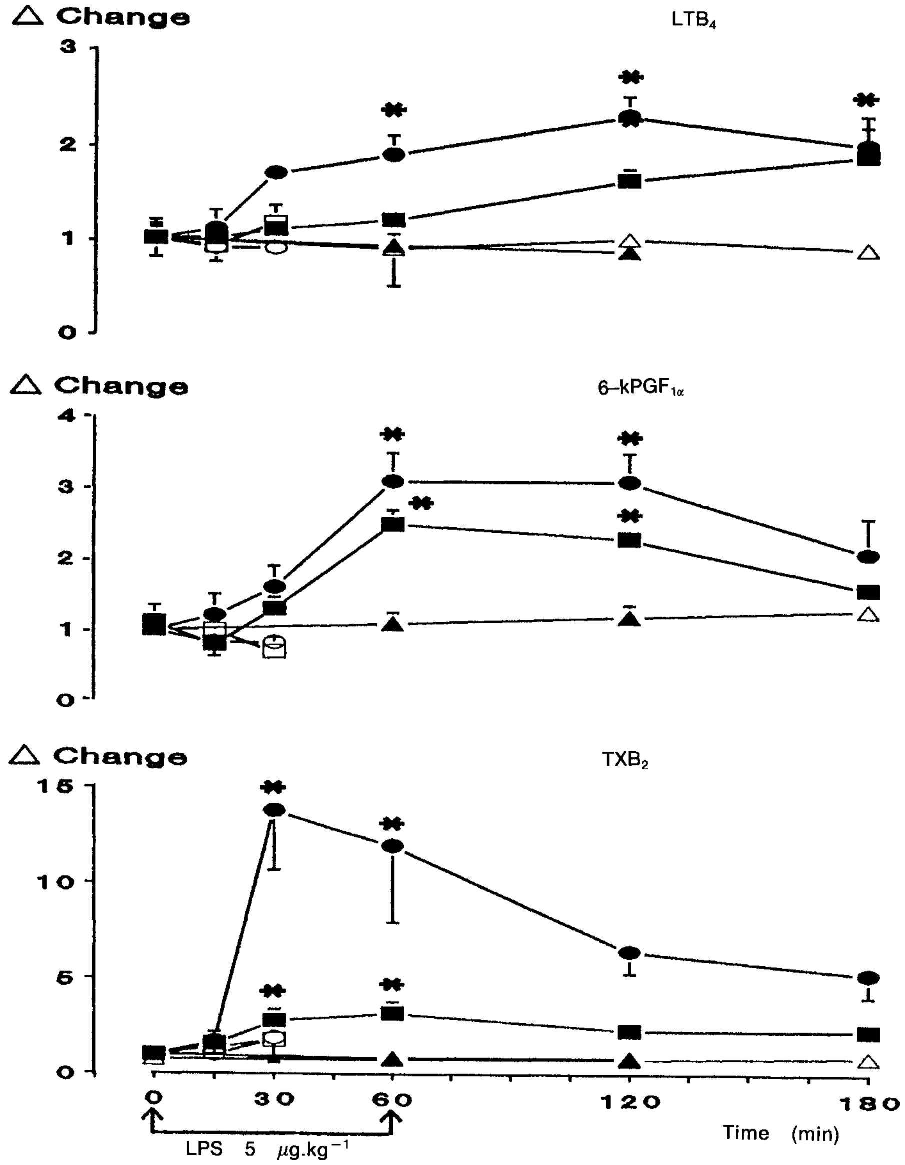

FIG. 2. Effect of BN 52021 pretreatment on plasma levels of eicosanoids in superior mesenteric vein in pigs receiving endotoxin (LPS) infusion into the superior mesenteric artery. Ordinate: logarithmic scale of changes in concentrations of leukotriene $B_4$ ($LTB_4$), 6-keto prostaglandin $F_{1\alpha}$ (6-keto $PGF_{1\alpha}$) and thromboxane $B_2$ ($TXB_2$). Other details as in Figure 1.

decreasing to the baseline at the end of the 3 h observation period; cardiac output first decreased rapidly (15–30 min after the start of LPS infusion) and, after a transient increase, decreased again gradually to about 35% of the baseline value (from the end of LPS infusion until the end of the observation period).

In BN 52021 pretreated survivors mean arterial blood pressure was preserved. At 180 min, mean arterial blood pressure in BN 52021 pretreated survivors ($n = 6$) was 105 ± 5 mmHg whereas in LPS treated survivors ($n = 16$) the MABP was 59 ± 5.5 mmHg, $p < 0.01$. BN 52021 did not affect the duration or the magnitude of the increase in mean portal venous pressure and mean pulmonary arterial pressure during or after LPS infusion. Though cardiac output was markedly reduced in BN 52021 pretreated survivors by the end of the experiment, it was still higher than in LPS infused survivors (1.5 ± 0.1 l/min, $n = 6$ *vs.* 1.0 ± 0.1 l/min, $n = 9$, $p < 0.05$).

*Mediator release (Figure 2):* Plasma concentration of $TXB_2$ in the superior mesenteric vein increased markedly, but only for a short time, during LPS infusion in survivors ($n = 5$). Similarly a transient, but less marked increase was detected in 6-keto $PGF_{1\alpha}$ concentrations of the superior mesenteric vein in survivors ($n = 5$). The increase in $TXB_2$ concentrations started 30 min earlier than in 6-keto $PGF_{1\alpha}$ elevation. In survivors, the plasma concentrations of $LTB_4$ also showed an increase in superior mesenteric vein ($n = 5$). However, $LTB_4$ production was still elevated, even when the

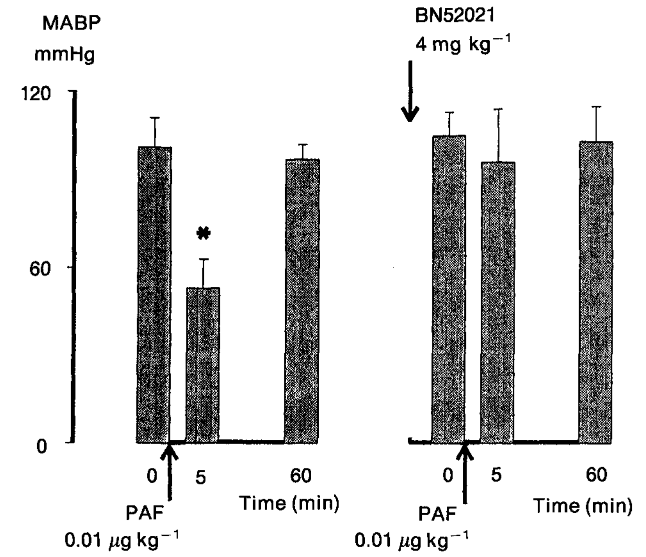

FIG. 3. Effect of BN 52021 (4 mg kg$^{-1}$) pretreatment on the hypotensive response to exogenous platelet activating factor (PAF) in anaesthetized pigs ($n = 4$). PAF (0.01 μg kg$^{-1}$) was injected into the superior mesenteric vein over 1 min. Arrows indicate the time for administration of either PAF or BN 52021, as appropriate. Other details as in Figure 1.

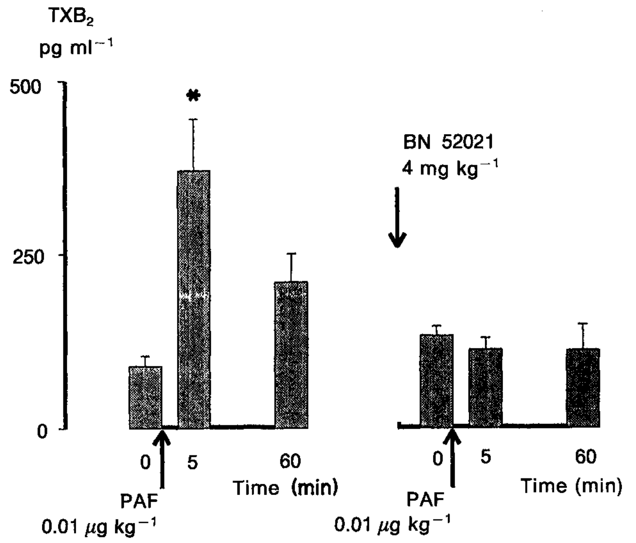

FIG. 4. Effect of BN 52021 (4 mg kg$^{-1}$) on the plasma level of thromboxane $B_2$ ($TXB_2$) in the superior mesenteric vein after administration of exogenous platelet-activating factor (PAF) in anaesthetized pigs ($n = 4$). PAF (0.01 μg kg$^{-1}$) was injected into the superior mesenteric vein over 1 min. Arrows indicate the time for administration of either PAF or BN 52021, as appropriate. Other details as in Figure 1.

concentrations of $TXB_2$ and 6-keto $PGF_{1\alpha}$ had returned to baseline. In contrast, no changes were found in eicosanoid concentrations in LPS infused non-survivors ($n = 5$).

In BN 52021 pretreated survivors the plasma concentration of $TXB_2$ was markedly reduced but remained slightly elevated ($n = 6$). PAF receptor antagonism had minimal effects on 6-keto $PGF_{1\alpha}$ and did not affect $LTB_4$ synthesis observed in LPS treated survivors. A correlation was found between mean pulmonary arterial blood pressure and the ratio of $TXB_2$ to 6-keto $PGF_{1\alpha}$ in BN 52021 pretreated survivors ($r_s$ 0.5542, $n = 25$, $p < 0.005$) as well as in LPS infused survivors ($r_s$ 0.5738, $n = 24$, $p < 0.001$). No changes were found in eicosanoids in BN 52021 pretreated non-survivors ($n = 7$).

*Effects of exogenous PAF and the effectiveness of BN 52021 treatment on exogenous PAF (Figures 3 to 5)*: Administration of 0.01 μg kg$^{-1}$ of PAF into the superior mesenteric vein over 1 min reduced mean arterial blood pressure from 101 ± 10 mmHg to 53 ± 10 mmHg in the absence, and from 105 ± 8 mmHg to 96 ± 18 mmHg in the presence, of BN 52021 (4 mg kg$^{-1}$) injected 5 min prior to PAF administration ($n = 4$, $p < 0.05$, Figure 3). This dose of PAF elevated plasma concentration of $TXB_2$ from 90 ± 15 pg ml$^{-1}$ to 370 ± 75 pg ml$^{-1}$ ($n = 4$, $p < 0.05$). This effect of PAF on thromboxane release was completely abolished by pretreatment with 4 mg kg$^{-1}$ of BN 52021 ($n = 4$, Figure 4). Administration of 0.01 μg kg$^{-1}$ of PAF into the superior mesenteric vein had no effect on plasma concentrations of 6-keto $PGF_{1\alpha}$ and $LTB_4$ at time points observed ($n = 4$, Figure 5).

### Discussion

The findings in the present study are: (a) the non-survivors following LPS infusion showed marked haemodynamic alterations and no changes in release of eicosanoids; (b) the animals surviving LPS infusion remained in a state of shock and presented a sequential release of eicosanoids; (c)

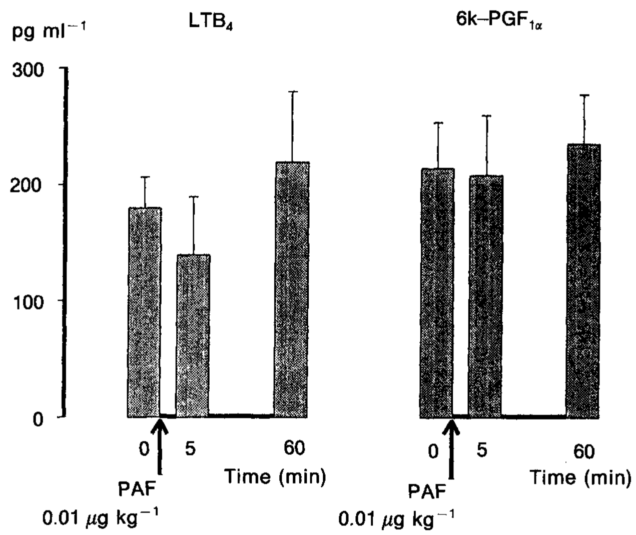

FIG. 5. Effect of exogenous platelet activating factor (PAF) on the plasma levels of leukotriene $B_4$ ($LTB_4$) and 6-keto prostaglandin $F_{1\alpha}$ (6-keto $PGF_{1\alpha}$) in the superior mesenteric vein in anaesthetized pigs ($n = 4$). PAF (0.01 μg kg$^{-1}$) was injected into the superior mesenteric vein over 1 min. Other details as in Figure 1.

treatment with PAF receptor antagonist BN 52021 (4 mg $kg^{-1}$) injected 5 min prior to LPS infusion, failed to exert any effect on the survival rate; (d) in BN 52021 pretreated survivors all circulatory parameters studies were improved, and thromboxane synthesis/release was markedly attenuated, whereas prostacyclin production was slightly modified and $LTB_4$ release was not affected; (e) exogenous administration of PAF (0.01 $\mu g\ kg^{-1}$) produced hypotension and an increase in the concentration of $TXB_2$ without any modification on the concentrations of 6-keto $PGF_{1\alpha}$ and $LTB_4$; (f) BN 52021 (4 mg $kg^{-1}$) pretreatment completely prevented the effects of exogenous PAF on blood pressure and thromboxane production.

Low amounts of LPS infused into the superior mesenteric artery provided a unique model for studying various changes induced by LPS infusion in surviving and non-surviving pigs as we have previously reported.[4,5,9] In the present work we were concerned to what extent death and endotoxic shock, induced by LPS infusion, are related to changes in lipid mediators activity.

Treatment with the PAF receptor antagonist BN 52021 5 min prior to the start of LPS administration did not prevent death and failed to modify the survival rate. This is understandable since the release of PAF has been observed only in surviving animals but not in non-survivors, thereby ruling out the role of endogenous PAF in causing death.[5] Similarly, no changes were observed in eicosanoid release for non-survivors; consequently, their role in the early death also seems to be negligible. The major change detected in non-survivors was a marked increase in TNF release that was not affected by BN 52021 treatment.[5,18] Thus it seems likely that death caused by LPS infusion is due to a marked increase in TNF release. This assumption is also supported by the findings that the amount of TNF released in survivors following LPS infusion was much less than in non-survivors.[18] The exact reason for the lack of PAF and eicosanoid release in non-survivors is not yet clear. It might be that TNF, released in large quantities during rapid death, has a destructive effect on the cells able to synthesize PAF and eicosanoids as our *in vivo* data and the *in vitro* data obtained by others indicate.[5,19]

In contrast to the lack of involvement of PAF and eicosanoids in LPS induced death, the present and our previous[4,5,9] results indicate that release of PAF and eicosanoids observed during LPS infusion may play a role in the development of endotoxic shock seen in survivors. Most of the critical events occurred at 30 min of endotoxaemia, a time point which coincides with the marked increase in concentrations of PAF[5] and $TXB_2$ in the superior mesenteric vein. It should be remembered that the peak in the release of PAF and thromboxane preceded the maximum in TNF production in LPS treated survivors. It might be that TNF, released in large quantities during rapid death, has a destructive effect on the cells able to synthesize eicosanoids.[9]

In addition, BN 52021 treatment significantly attenuated both the magnitude and duration of haemodynamic changes in survivors. Mean arterial blood pressure remained stable, while cardiac output decreased but remained still higher than in LPS treated survivors. On the other hand, BN 52021 had no effect on the elevated portal and pulmonary pressures, while the thromboxane synthesis/release was markedly attenuated, indicating that mediators other than PAF and thromboxane might be involved. Another explanation may be that the balance between thromboxane and prostacyclin might be more important than the change in thromboxane and prostacyclin levels in itself. Considerable evidence exists that the thromboxane to prostacyclin ratio is a critical factor in coronary artery disease[20] as well as in mesenteric ischaemia/reperfusion-induced shock[21] and endotoxic shock.[4] However, it should be kept in mind that the concentrations at which these eicosanoids exert opposite effects are unknown. Moreover, it is also important that the concentrations of $TXB_2$ and 6-keto $PGF_{1\alpha}$ do not reflect exactly the release of thromboxane and prostacyclin, but only the net result of their release, degradation and renal and hepatic clearance.[3] Nevertheless, present results demonstrate a correlation between $TXB_2$ to 6-keto $PGF_{1\alpha}$ ratio and pulmonary arterial pressure while this correlation was not observed when absolute concentrations of these compounds were used. One should remember that in the present investigations BN 52021 altered only slightly the prostacyclin production while markedly attenuated the thromboxane production resulting in an imbalance between thromboxane and prostacyclin in favour of prostacyclin, which is thought to be beneficial in different shock states.[22] The exact benefits of a PAF receptor antagonist on endotoxic shock are unclear; however, present results indicate that they may be related to the effects of BN 52021 on prostanoid synthesis.

The rationale for evaluating the effects of a PAF receptor antagonist on the eicosanoid products of arachidonic acid is that these lipid mediators (i.e. PAF and eicosanoids) have been shown to interact with each other under several conditions.[13–16] The injection of PAF into an isolated gastric perfusion model significantly increased the production of thromboxane and prostacyclin which resulted in vasoconstriction.[23] The PAF receptor antagonist, BN 52021, not only reduced the vasoconstriction, but also significantly inhibited PAF-induced eicosanoid release.[23] Acute circulatory collapse in dogs from exogenous PAF administration is associated with the release of thromboxane and prostacyclin.[24] However, in the present experiment the hypotensive response to exogenous PAF, injected into the superior mesenteric vein, was only associated with the increase in plasma concentration of $TXB_2$. No changes were observed in plasma concentrations of 6-keto $PGF_{1\alpha}$ and $LTB_4$ following exogenous PAF administration. This discrepancy might be attributable to either species differences or differences in the doses and route of administration of PAF (i.e. injection into the mesenteric vein in the present study *vs.* injection into the femoral or gastric vessels in the previous experiments).[23,24] The differences in the doses used may be one of the most important explanations for the discrepancy between the effects of exogenous PAF. Namely, low concentrations of PAF ($10^{-13}$–$10^{-10}$ M) induce the conversion of arachidonic acid into mainly prostanoids, while higher concentrations of PAF ($10^{8}$–$10^{-5}$ M) increase leukotriene production via a biphasic change in cyclic AMP level.[25] In the present study the peak concentrations of PAF in the mesenteric blood can be calculated as about 1000–1300 pg $ml^{-1}$.[5] These values are in the same order of magnitude as those measured at 30 min of endotoxaemia[5] suggesting that the amount of PAF formed during LPS infusion might be sufficient for causing the observed changes in thromboxane release. The maximum release of PAF and thromboxane following LPS infusion occurred at the same time and both preceded the peak in 6-keto-$PGF_{1\alpha}$ and $LTB_4$ concentrations, suggesting a dissociation in eicosanoid release evoked by administration of exogenous PAF. Further evidence for dissociation in eicosanoid release following PAF injection might be the results with BN 52021. At a concentration of 4 mg $kg^{-1}$ this drug inhibited markedly thromboxane synthesis following endotoxin in the experiments described, while PAF receptor antagonism had minimal effects on prostacyclin and leukotriene synthesis. These observations of pig endotoxaemia are similar to those reported in rat and dog endotoxaemia.[11,12] The exact reason, however, for the blocking effect of BN 52021 on prostanoid production is not yet clear. It is highly unlikely that BN 52021 acted as a cyclooxygenase inhibitor, since it failed to affect prostanoid formation in sham operated pigs. Whether PAF and/or BN 52021 could modify phospholipase $A_2$ activity remains to be examined. Taken together, the data in the present study suggest that PAF stimulates the release of thromboxane, and PAF receptor antagonism has its major effect on thromboxane production in endotoxaemia.

The findings of the present study give further support to the notion that PAF and eicosanoids may have an important interaction during endotoxic shock.[1,11,12] In the past few years, it has become increasingly apparent that a relationship exists between PAF and eicosanoids.[10] Once released, PAF and prostanoids may exert a wide range of biological activities, like hypotension, haemocontractions and activation of various cells,[24,26,27] which, in turn, release other vasoactive mediators.[27] Our observation that the PAF receptor antagonist prevented the prostanoid release is consistent and supports the suggestion that PAF may serve as a focal distributor of eicosanoids.[3]

In conclusion, the present data suggest that prostanoid formation is secondary to PAF release and prostanoids may mediate and/or modulate the haemodynamic effeccts of PAF during LPS infusion induced shock. However, their role in early death seems to be negligible. It appears that mediators other than PAF and eicosanoids are also involved in the development of endotoxic shock.

### References

1. Braunwald E, Isselbacher KJ, Petersdorf RG, Wilson RG, Martin JB, Fauci AS. *Harrison's Principles of Internal Medicine*, 11th ed. Edn. New York: McGraw-Hill Company, 1987; 474.
2. Zimmerman JJ. Therapy for overwhelming sepsi-Clues for treating disease and not just the symptoms. *Crit Care Med* 1990; **18**: 118–119.
3. Lefer AM. Significance of lipid mediators in shock states. *Circ Shock* 1989; **27**: 3–12.
4. Mózes T, Zijlstra FJ, Heiligers JPC, Saxena PR, Bonta IL. Sequential release of eicosanoids during endotoxin-induced shock in anaesthetized pigs. *Prostaglandins Leukotrienes and Essential Fatty Acids* 1991; **42**: 209–216.
5. Mózes T, Heiligers JPC, Tak CJAM, *et al.* Platelet activating factor of one of the mediators involved in endotoxic shock in pigs. *J Lipid Mediators* 1991; **4**: 309–326.
6. Ball HA, Cook JA, Wise WC, Halushka PV. Role of thromboxane, prostaglandins and leukotrienes in endotoxic and septic shock. *Intensive Care Med* 1986; **12**: 116–126.
7. Mózes T, Wieszt E, Zahajszky T. Role of $PGF_{2\alpha}$ in the superior mesenteric artery-induced shock. *J Surg Res* 1989; **47**: 476–481.
8. Filep J, Braquet P, Mózes T. Interactions between platelet-activating factor and prostanoids during mesenteric ischemia-reperfusion-induced shock in the anaesthetized dog. *Circ Shock* 1991; **35**: 1–8.
9. Mózes T, Zijlstra FJ, Heiligers JPC, *et al.* Sequential release of tumour necrosis factor, platelet activating factor and eicosanoids during endotoxic shock in anaesthetized pigs; protective effects of indomethacin. *Br J Pharmacol* 1991; **104**: 691–699.
10. Chilton FH, Cluzel M, Triggiani M. Recent advances in our understanding of the biochemical interactions between platelet-activating factor and arachidonic acid. *Lipids* 1991; **26**: 1021–1027.
11. Fletcher JR, DiSimone AG, Earnest MA. Platelet activating factor receptor antagonist improves survival and attenuates eicosanoid release in severe endotoxemia. *Ann Surg* 1990; **211**: 313–316.
12. Moore MJ, Earnest MA, DiSimone AG, Abumrad NN, Fletcher JR. A PAF receptor antagonist, BN 52021, attenuates thromboxane release and improves survival in lethal canine endotoxemia. *Circ Shock* 1991; **35**: 53–59.
13. Feuerstein G, Boyd LM, Ezra D, Goldstein RE. Effect of platelet-activating factor on coronary circulation of the domestic pig. *Am J Physiol* 1983; **246**: H466–H471.
14. Chung KF, Aizawa H, Leikauf GD, Ueki IF, Evans TW, Nadel JA. Airway hyperresponsiveness induced by platelet-activating factor. Role of thromboxane generation. *J Pharmacol Exp Ther* 1986; **236**: 580–584.
15. Voelkel NF, Worthen S, Henson PM, Murphy RC. Nonimmunological production of leukotrienes induced by platelet-activating factor. *Science* 1982; **218**: 286–288.
16. Hsueh W, Gonzales-Crussi F, Arroyave JL. Platelet-activating factor-induced bowel necrosis. An investigation of secondary mediators in its pathogenesis. *Am J Pathol* 1986; **122**: 231–239.
17. Zijlstra FJ, Vincent JE. Determinations of leukotrienes and prostaglandins in $^{14}C$ arachidonic acid labelled human lung tissue by high-performance liquid chromatography and radioimmunoassay. *J Chromatogr* 1984; **311**: 39–50.
18. Mózes T, Ben-Efraim S, Tak CJAM, Heiligers JPC, Saxena PR, Bonta IL. Serum levels of tumor necrosis factor determine the fatal or non-fatal course of endotoxic shock. *Immunol Lett* 1991; **27**: 157–162.
19. Braquet P, Paubert-Braquet M, Bourgain RH, Bussolino F, Hosford D. PAF/cytokine auto-generated feedback networks in microvascular immune injury: consequences in shock, ischemia and graft rejection. *J Lipid Mediators* 1989; **1**: 75–112.

20. Lefer AM. Prostacyclin, high density lipoproteins, and myocardial ischemia. *Circulation* 1990; **81**: 2013–2015.
21. Mózes T, Wieszt E. Intestinal and arterial plasma thromboxane and prostacyclin levels in shock: effects of indomethacin. *J Surg Res* 1989; **47**: 482–486.
22. Lefer AM. Role of prostaglandins and thromboxanes in shock states. In: Altura BM, Lefer AM, Schummer W, eds. *Handbook of Shock and Trauma.* New York: Raven, 1983; 366–367.
23. Dembinska-Kiec A, Peskar BA, Muller MK, Peskar BM. The effects of platelet-activating factor on flow rate and eicosanoid release in the isolated perfused rat gastric vascular bed. *Prostaglandins* 1989; **37**: 69–91.
24. Bessin P, Bonnet J, Apfel D, *et al.* Acute circulatory collapse caused by platelet-activating factor (PAF-acether) in dogs. *Eur J Pharmacol* 1983; **86**: 403–411.
25. Beusenberg FD, van Schaik A, van Amsterdam JGC, Bonta IL. Involvement of eicosanoids in platelet activating factor-induced modulation of adenylyl cyclase activity in alveolar macrophages. *J Lipid Med* 1991; **3**: 301–310.
26. Sanchez-Crespo M, Alonso F, Inarrea P, Alvarez V, Egido J. Vascular actions of synthetic PAF-acether (a synthetic platelet-activating factor) in the rat; evidence for platelet independent mechanism. *Immunopharmacology* 1982; **4**: 173–185.
27. Braquet P, Touqui L, Shen TY, Vargaftig BB. Perspective in platelet-activating factor research. *Pharmacol Res* 1987; **39**: 97–145.

ACKNOWLEDGEMENTS. This research was supported by grants from the Hungarian Academy of Sciences (OTKA 1115), Erasmus University Foundation (Stichting Universiteitsfonds Rotterdam), the Emil Starkenstein Foundation and l'Institut Henri Beaufort, obtained through courtesy of Dr P. Braquet. We are grateful to Mrs I. M. Garrelds for performing eicosanoid measurements and to Mr C. J. A. M. Tak for drawing the figures.

**Received 18 March 1992;
accepted in revised form 31 March 1992**
